# Targeting Kynurenine Synthesis Attenuated Cigarette Smoke‐Induced COPD‐Like Lung Injury Driven by TALDO1‐Mediated Metabolic Reprogramming

**DOI:** 10.1002/advs.78795

**Published:** 2026-11-11

**Authors:** Wenjing Wang, Mindan Wu, Yuanlan Yang, Zemin Chen, Jiawei Yan, Xuan Jin, Yachao Cui, Feng Zhang, Haiqing Li, Wensheng Chen, Yumin Zhou, Leqing Zhu, Wei Tu, Pixin Ran

**Affiliations:** ^1^ State Key Laboratory of Respiratory Diseases National Clinical Research Center for Respiratory Diseases Guangzhou Institute of Respiratory Health the First Affiliated Hospital of Guangzhou Medical University Guangzhou Guangdong China; ^2^ Guangzhou National Laboratory Guangzhou Guangdong China; ^3^ GMU‐GIBH Joint School of Life Sciences Guangzhou Medical University Guangzhou China

**Keywords:** chronic obstructive pulmonary disease, L‐kynurenine, mitochondrial injury, TALDO1

## Abstract

Chronic obstructive pulmonary disease (COPD) is a devastating condition characterized by airflow limitation, inflammation, and structural lung damage. Metabolic dysregulation is recognized as a critical feature, but the key metabolic drivers remain unclear. This study identifies transaldolase1 (TALDO1) as a previously unrecognized metabolic regulator in cigarette smoke (CS)‐induced COPD pathogenesis. TALDO1 is markedly upregulated in airway epithelial cells by CS exposure. Elevated TALDO1 promotes mitochondrial dysfunction, whereas its genetic knockdown attenuates CS‐induced lung function decline and pulmonary inflammation. Mechanistically, TALDO1 activation redirects tryptophan metabolism toward the kynurenine pathway, leading to L‐kynurenine accumulation. Genetic or pharmacological inhibition of this pathway significantly alleviates mitochondrial damage, while L‐kynurenine supplementation exacerbates injury. Treatment with the IDO inhibitor Epacadostat ameliorates CS‐induced lung impairment and inflammation in vivo. These findings define a TALDO1‐kynurenine‐mitochondrial injury axis linking airway epithelial metabolic reprogramming to COPD, providing translational evidence for targeting this pathway as a therapeutic strategy.

## Graphical abstract

Cigarette smoke induces airway epithelial TALDO1 upregulation and L‐kynurenine accumulation, driving mitochondrial injury with elevated mtROS, reduced Δψm, ATP, and OCR. This links to lung inflammation, alveolar destruction, and lung function damage, all attenuated by Epacadostat or Taldo1 reduction. The study highlights TALDO1/kynurenine‐mediated mitochondrial dysfunction in smoking‐related lung pathology, suggesting a potential therapeutic target.

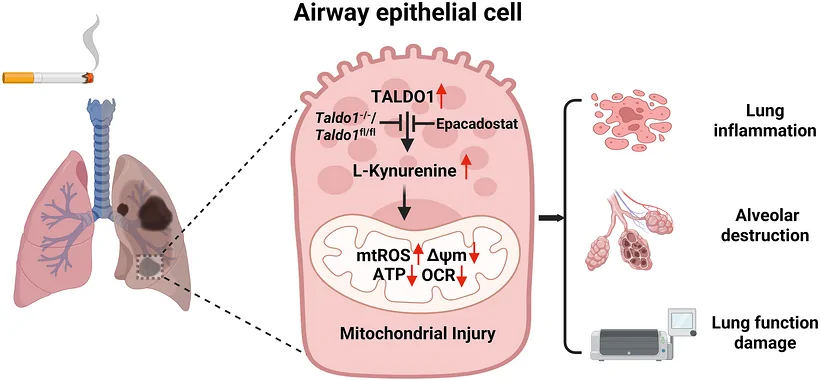

## 1 Introduction

Chronic obstructive pulmonary disease (COPD) is a leading cause of morbidity and mortality worldwide [1, 2, 3], imposing a substantial clinical and socioeconomic burden [4, 5]. Cigarette smoke (CS) exposure remains the primary risk factor for COPD and contributes to persistent airway inflammation, epithelial injury, and progressive lung function decline. Given that the small airways undergo irreversible destruction at the early stages of disease and no effective therapies are currently available to halt this progression [6, 7], understanding the pathogenic mechanisms underlying early small‐airway lesions is crucial for improving COPD management. In recent years, mitochondrial dysfunction has emerged as a pathological feature driving epithelial injury, metabolic maladaptation, oxidative stress, and premature cellular senescence in COPD [8, 9, 10]. However, despite growing recognition of the importance of mitochondrial impairment in COPD pathogenesis, the upstream metabolic regulators linking CS exposure to disrupted mitochondrial homeostasis remain inadequately defined.

Metabolic reprogramming has recently attracted considerable interest as a hallmark of chronic respiratory diseases, including COPD [11, 12, 13]. Among the various metabolic pathways involved, the tryptophan‐kynurenine pathway plays a critical role in immune regulation, oxidative balance, and inflammation [14, 15]. Elevated kynurenine levels have been reported in chronic inflammatory disorders [16, 17], and disruption in kynurenine metabolism was associated with mitochondrial dysfunction [18], reactive oxygen species (ROS) accumulation [18, 19], and impaired macrophage function [20, 21]. Moreover, accumulating evidence suggests that kynurenine pathway metabolites can directly influence mitochondrial and cellular homeostasis [22, 23, 24]. CS has also been reported to perturb amino acid metabolism [25, 26]; however, the specific remodeling of kynurenine metabolism during COPD progression and its potential role in CS–induced mitochondrial dysfunction remain largely unexplored.

Airway epithelial dysfunction in COPD is closely associated with metabolic reprogramming, redox imbalance, and mitochondrial injury, yet the metabolic regulators connecting these processes remain poorly characterized. To identify candidate regulators involved in COPD‐associated airway epithelial dysfunction, transcriptomic screening of airway‐derived samples was performed and identified transaldolase 1 (TALDO1), a key enzyme of the non‐oxidative pentose phosphate (PPP) pathway, as a significantly upregulated candidate in COPD. TALDO1 catalyzes the reversible transfer of three‐carbon units within the non‐oxidative PPP and contributes to the redistribution of carbon intermediates between the PPP and glycolysis. Although TALDO1 does not directly generate NADPH, alterations in TALDO1 activity may influence carbon recycling through the PPP and thereby affect cellular metabolic and redox homeostasis [27]. Dysregulated TALDO1 expression has been associated with oxidative stress [28], metabolic instability [29, 30], and cell injury [31] in several diseases. Together with our preliminary observations suggesting a potential relationship between TALDO1 and mitochondrial homeostasis, these findings prompted further investigation of TALDO1 in COPD.

Despite these observations, the role of TALDO1 in chronic airway inflammation and metabolic remodeling in COPD remains incompletely understood. In particular, whether TALDO1 links CS‐induced epithelial metabolic reprogramming to altered tryptophan–kynurenine metabolism and mitochondrial dysfunction has not been established. Therefore, the present study investigated the regulation and functional significance of TALDO1 in response to CS exposure and examined its contribution to metabolic reprogramming and mitochondrial dysfunction in COPD. Our findings identify TALDO1 as a previously unrecognized metabolic regulator that promotes kynurenine accumulation and exacerbates epithelial mitochondrial injury under CS exposure. Pharmacological inhibition of IDO1 with Epacadostat (Epa) reduced kynurenine accumulation and attenuated CS‐induced mitochondrial dysfunction and COPD‐related pathological changes in mice. Our findings uncover a previously unrecognized TALDO1‐kynurenine‐mitochondrial axis linking metabolic perturbation to epithelial injury and suggest that this pathway may provide new metabolic targets for therapeutic intervention in COPD.

## 2 Results

### 2.1 *TALDO1* is Upregulated in Airway Epithelial Cells of COPD Patients and CS‐Induced Model In Vitro and In Vivo

To identify candidate regulators involved in COPD‐associated airway epithelial dysfunction, differential expression analysis was performed using the airway‐derived transcriptomic dataset GSE10006. This analysis revealed a panel of dysregulated genes, among which TALDO1 was significantly upregulated in COPD airway samples (Figure S1A), prompting its selection for further investigation. We next examined the reproducibility of this finding across independent airway epithelial transcriptomic datasets. Analysis of GSE10006 and GSE5060 confirmed increased *TALDO1* expression in smokers and COPD patients compared with non‐smokers (Figure 1A). The clinical relevance of TALDO1 was further evaluated in lung tissues from 110 COPD patients and 37 healthy controls in the GSE76925 dataset. We found that higher *TALDO1* expression was associated with lower forced expiratory volume in 1 s as a percent predicted (FEV1% Predicted) and a lower FEV1/forced vital capacity (FEV1/FVC) ratio (Figure 1B). The two visually apparent groupings in Figure 1B mainly reflected the separation between the COPD and healthy control groups and were largely attributable to differences in pulmonary function. We then measured *TALDO1* expression in primary human airway epithelial cells from COPD patients and healthy controls. Both *TALDO1* mRNA and TALDO1 protein levels were markedly increased in cells from COPD patients (Figure 1C,D). Meanwhile, 3 and 6 months of CS exposure significantly increased Taldo1 expression in mouse lungs (Figure 1E, Figure S1B,C). Immunofluorescent staining further showed that Taldo1 expression co‐localized with the airway epithelial marker SCGB1A1 (Figure 1F), supporting its epithelial localization.

**FIGURE 1:**
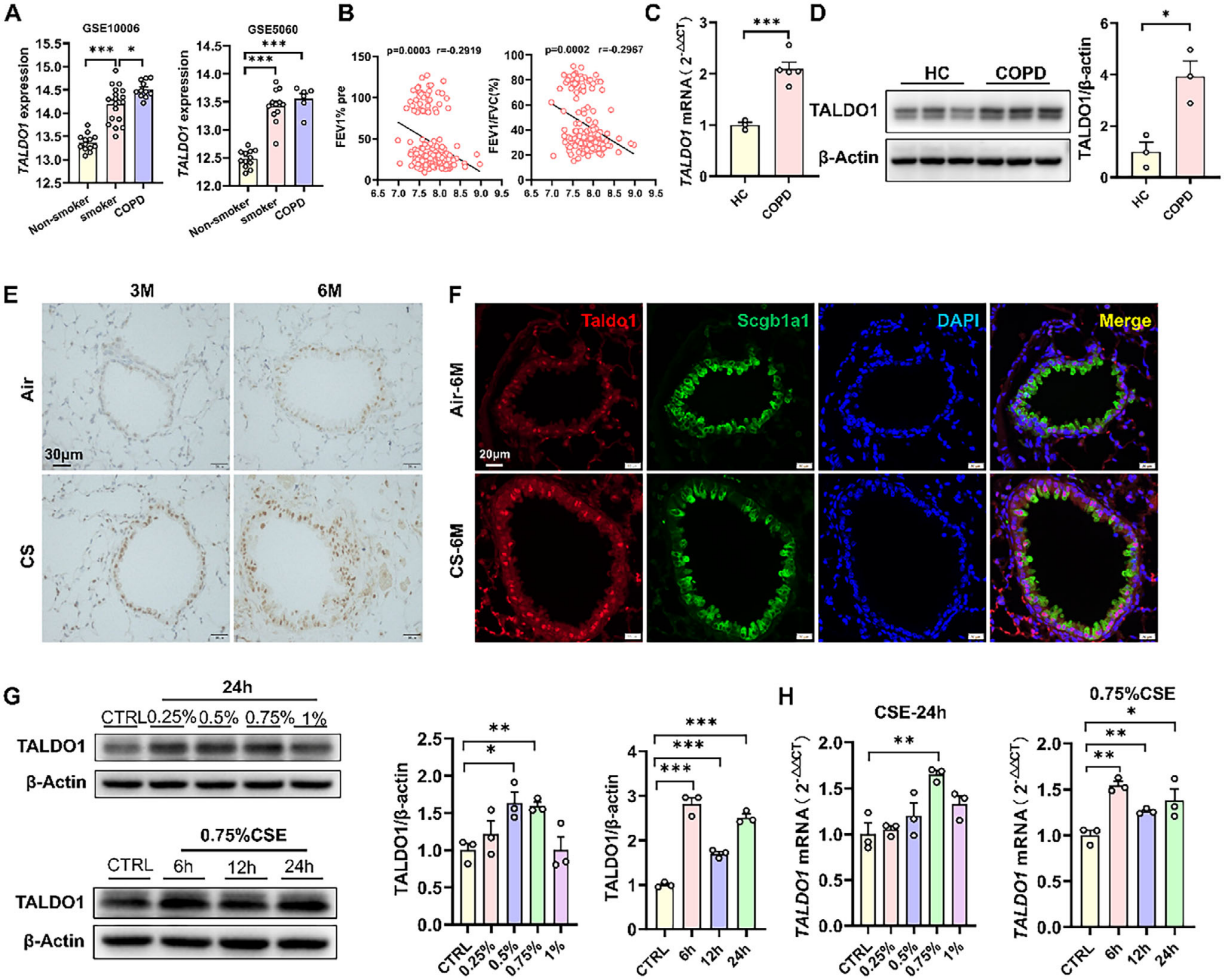
TALDO1 is upregulated in airway epithelial cells of COPD patients and the CS‐induced model. (A) *TALDO1* mRNA levels in airway epithelial cells from non‐smokers, smokers, and COPD patients were analyzed using public datasets GSE10006 and GSE5060. (B) Correlation of *TALDO1* expression with FEV1% predicted (FEV1%pre) and FEV1/FVC ratio was performed using data from GSE76925. (C, D) *TALDO1* expression in airway epithelial cells from COPD patients and healthy controls was measured by RT‐PCR (C) and Western blot (D). (E) Immunohistochemical images of Taldo1 in lung sections from mice treated with or without CS for 3 or 6months. (F) Co‐staining of immunofluorescent staining for *Taldo1* and *SCGB1A1* in lung tissues of mice exposed to CS for 6 months. (G, H) Western blot and mRNA expression analyses of *TALDO1* in HBE cells exposed to different concentrations and durations of CSE. Data are presented as means ± SEMs. ^*^ *p* < 0.05; ^**^ *p* < 0.01; ^***^ *p* < 0.001.

To determine whether CS directly induced TALDO1 expression in airway epithelial cells, we treated human bronchial epithelial (HBE) cells with different doses of cigarette smoke extract (CSE) for 24 h and found that 0.5% and 0.75% CSE significantly increased TALDO1 expression compared with untreated cells (Figure 1G,H, left panel). Time‐course experiments further showed that treatment with 0.75% CSE significantly elevated TALDO1 expression at 6, 12, and 24 h (Figure 1G,H, right panel). Taken together, these findings indicated that TALDO1 is consistently upregulated in airway epithelial cells from patients with COPD, as well as in CS‐induced models both in vivo and in vitro.

### 2.2 Airway Epithelial‐Specific Deletion of *TALDO1* Attenuates CS‐Induced Lung Function Impairment, Airway Inflammation, and Pulmonary Remodeling

To explore the functional role of *TALDO1* in COPD, we established a chronic CS‐induced mouse model using airway epithelial‐specific *Taldo1* conditional knockout mice (*Scgb1a1*‐Cre^+^; *Taldo1* ^fl/fl^_,_ hereafter *Taldo1* ^fl/fl^ Cre), following the protocol illustrated in Figure 2A. Successful conditional deletion was confirmed by PCR genotyping and immunofluorescent staining (Figure S2B,C). Immunofluorescence analysis further showed that Taldo1 expression is selectively reduced in SCGB1A1^+^ airway epithelial cells, without detectable changes in macrophages and alveolar type II epithelial cells (Figure S2D).

**FIGURE 2:**
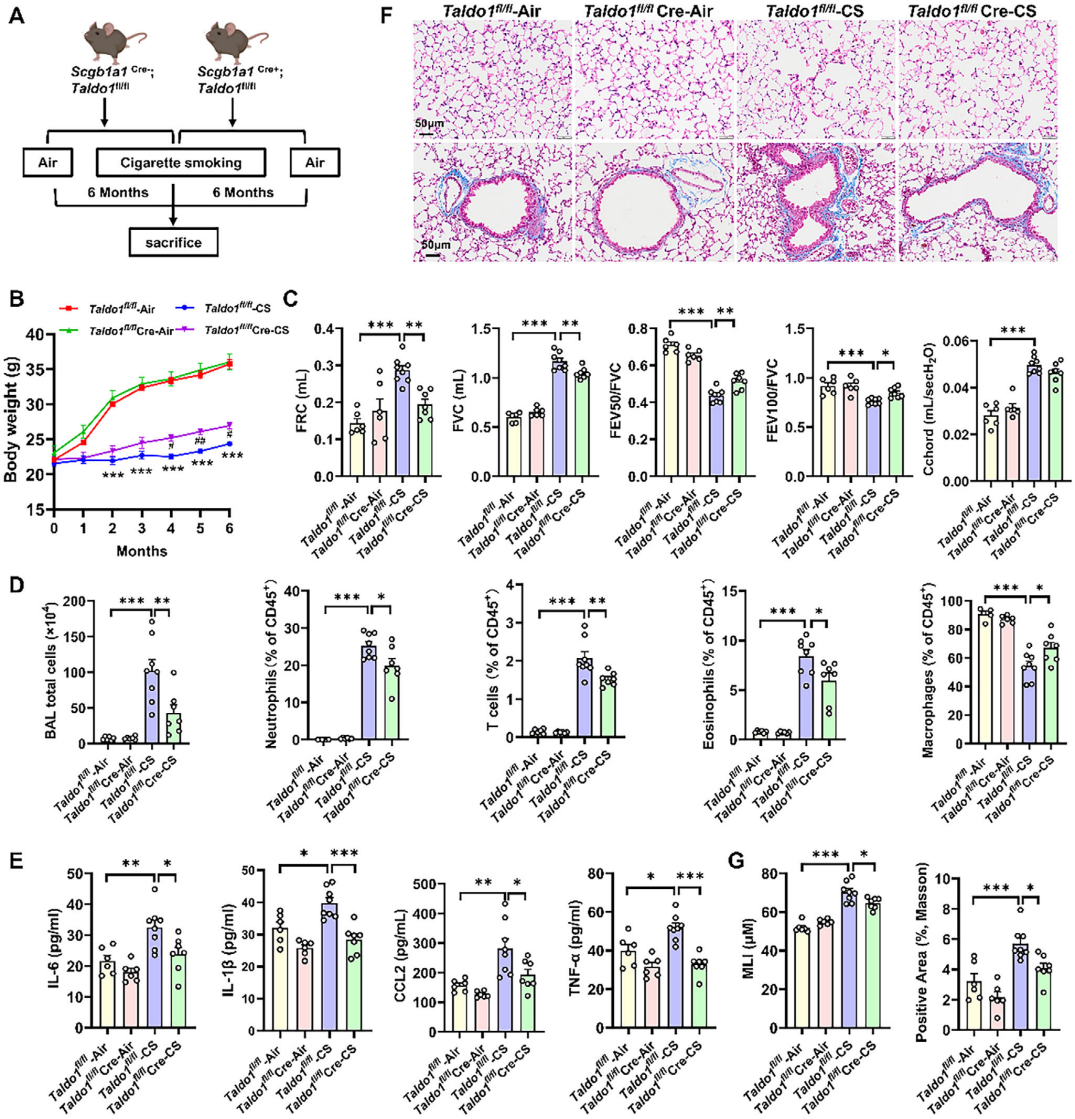
Airway epithelial‐specific knockout of *Taldo1* improves CS‐induced lung function decline and airway inflammation in mice. (A) Protocol for CS‐induced mouse model. (B) Body weight measurement within 6 months with/without CS exposure. (C) Pulmonary function analysis in the mouse model. (D) BAL fluid analysis of total cell counts and differential cells by flow cytometry. (E) ELISA measurement of cytokines in BAL fluid. (F) H&E (upper panel) and Masson's trichrome (lower panel) staining for mouse lung tissues. (G) Quantitative analysis of MLI and peribronchial collagen deposition, respectively. *n* = 6–8 per group. Data are presented as means ± SEMs. ^*^ *p* < 0.05; ^**^ *p* < 0.01; ^***^ *p* < 0.001.

As shown in Figure 2B, CS exposure significantly reduced body weight in *Taldo1* ^fl/fl^ mice compared with air‐exposed controls, whereas airway epithelial‐specific deletion of *Taldo1* attenuated CS‐induced body weight loss. Lung function revealed that 6 months of CS exposure markedly increased FVC, chord compliance (Cchord) and functional residual capacity (FRC), while decreasing FEV50/FVC and FEV100/FVC in *Taldo1* ^fl/fl^ mice relative to air‐exposed controls (Figure 2C). These CS‐induced abnormalities were significantly attenuated in *Taldo1* ^fl/fl^ Cre mice, as evidenced by lower FRC and FVC, and higher FEV50/FVC and FEV100/FVC compared with CS‐exposed *Taldo1* ^fl/fl^ mice (Figure 2C).

Analysis of bronchoalveolar lavage (BAL) fluid further showed a pronounced inflammatory response following CS exposure. CS exposure significantly increased the total cell numbers as well as the percentage of neutrophils, T cells, and eosinophils among CD45^+^ cells in *Taldo1* ^fl/fl^ mice compared with air controls, whereas the relative proportion of alveolar macrophages was decreased (Figure 2D). Notably, despite their reduced relative frequency, the absolute number of alveolar macrophages was significantly increased following CS exposure (Figure S3A), indicating that the lower macrophage percentage reflected a shift in the overall immune‐cell composition rather than a reduction in macrophage abundance. Importantly, airway epithelial‐specific deletion of *Taldo1*markedly reduced CS‐induced total cell accumulation and attenuated the changes in inflammatory immune‐cell composition (Figure 2D). Consistently, ELISA showed that CS exposure increased BAL fluid levels of IL‐6, IL‐1β, CCL2 and TNF‐α in *Taldo1* ^fl/fl^ mice, whereas *Taldo1* airway epithelial‐specific deficiency significantly reduced CS‐induced increases in IL‐6, IL‐1β, CCL2 and TNF‐α (Figure 2E). Similarly, mRNA expressions of *Cxcl1, Cxcl2, Ccl2*, *Tnf‐α, Mmp12* and *Mmp13* were increased in lung tissues following CS exposure and were attenuated by airway epithelial‐specific *Taldo1* deletion (Figure S3B).

Histopathological analysis further demonstrated that CS exposure caused pronounced pulmonary structural alterations, including destruction of alveolar architecture, enlargement of alveolar airspaces as reflected by an increase in mean linear intercept (MLI), and airway remodeling assessed by Masson's trichrome staining (Figure 2F,G, Figure S3C). These pathological changes were substantially alleviated in *Taldo1* ^fl/fl^ Cre mice compared with *Taldo1* ^fl/fl^ mice induced by CS exposure. Consistent protective effects were also observed in *Taldo1* ^−/−^ mice (Figure S4). Together, these results indicate that airway epithelial‐specific loss of *Taldo1* ameliorates CS‐induced lung dysfunction, emphysematous changes, airway inflammation, and airway remodeling, highlighting a protective role for epithelial TALDO1 deletion in CS‐induced COPD‐like lung injury.

### 2.3 TALDO1 Mediates CS‐Induced Mitochondrial and Metabolic Dysfunction in Airway Epithelial Cells

To determine whether mitochondrial dysfunction is involved in COPD and whether TALDO1 contributes to this process, we first assessed mitochondrial and metabolic function in primary human airway epithelial cells and CSE‐treated HBE cells. Compared to cells from healthy individuals, airway epithelial cells from COPD patients showed reduced ATP levels and mitochondrial membrane potential, together with increased mitochondrial reactive oxygen species (mtROS) production (Figure S5A–C). Consistently, exposure of HBE cells to CSE induced a time‐dependent increase in mtROS and a progressive decrease in mitochondrial membrane potential (Figure S5D,E). Metabolic flux analysis further demonstrated that CSE induced a broad impairment of cellular metabolic capacity. Seahorse mitochondrial stress test revealed that cells exposed to CSE for 12 and 24 h exhibited reduced basal respiration and spare respiratory capacity (Figure S5F). Glycolytic rate analysis similarly showed reduced proton efflux rate (PER), characterized by a reduction in both basal glycolysis and compensatory glycolysis (Figure S5G). These findings indicate that CSE exposure damages not only mitochondrial oxidative phosphorylation but also the glycolytic capacity of airway epithelial cells.

We next investigated whether TALDO1 was associated with these metabolic abnormalities. Airway epithelial cells from the GSE10006 dataset were stratified according to *TALDO1* expression, followed by GO enrichment analysis. As shown in Figure 3A, *TALDO1* was found to be associated with biological processes related to regulation of mitochondrial membrane potential, oxidative stress, apoptosis, and inflammatory signaling. Consistent with these transcriptomic associations, transmission electron microscopy of airway epithelial cells from our mouse model showed that CS exposure induced marked mitochondrial ultrastructural abnormalities in *Taldo1^fl/fl^* mice, including matrix swelling and disruption or loss of mitochondrial cristae. These mitochondrial structural abnormalities were substantially alleviated in airway epithelial‐specific *Taldo1‐*deficient *mice* after CS exposure (Figure 3B).

**FIGURE 3:**
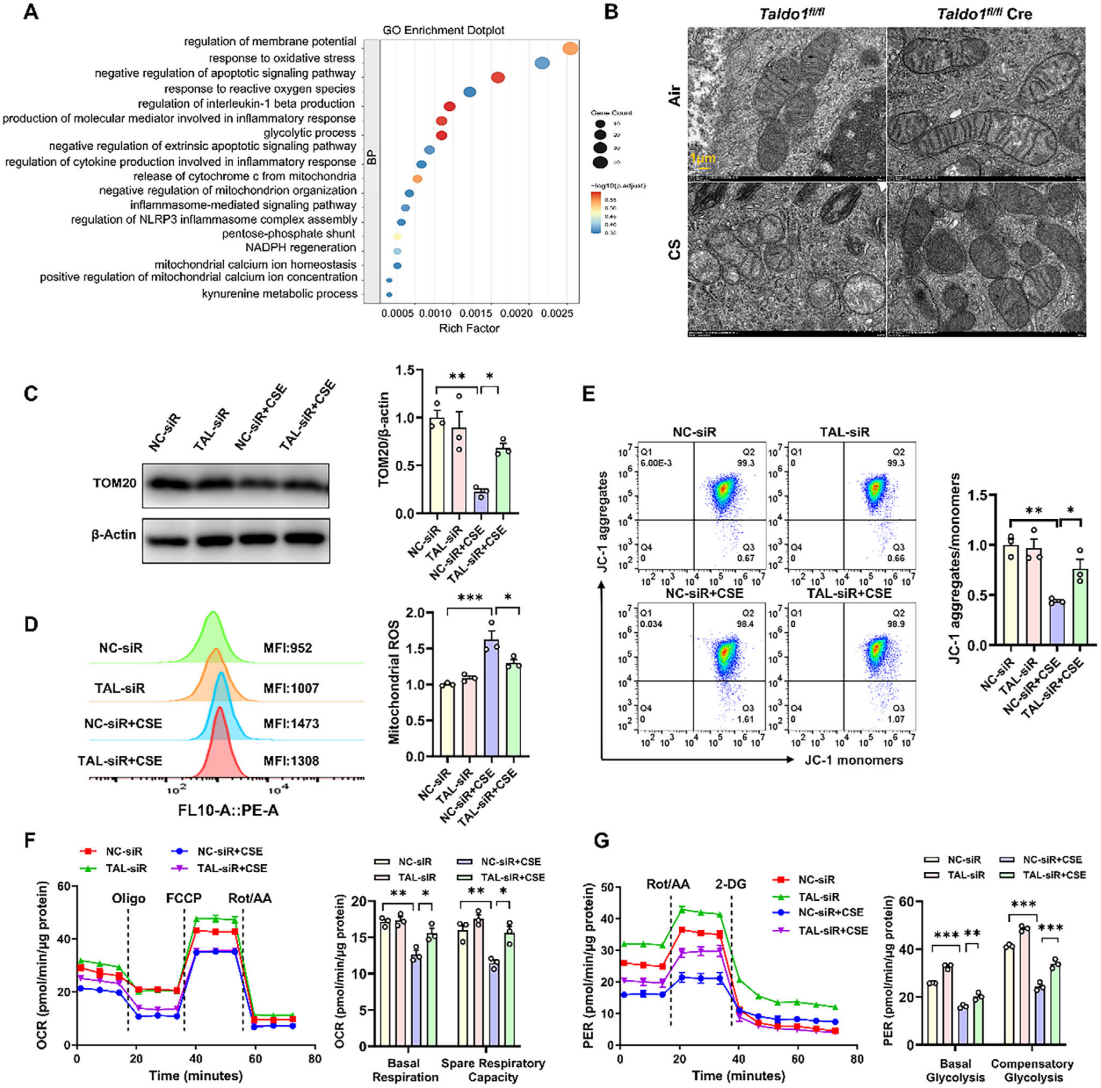
Taldo1‐specific knockout attenuates CS‐induced mitochondrial and metabolic dysfunction in airway epithelial cells. (A) GO enrichment analysis of primary airway epithelial cells from COPD patients in the GSE10006 dataset, stratified by *TALDO1* expression (high vs low). (B) Representative images of mitochondrial ultrastructure in airway epithelial cells from mouse lung tissues. (C–G) Analysis of TOM20 expression (C), mtROS production (D), mitochondrial membrane potential (E), OCR (F), and PER (G) in HBE cells transfected with *TALDO1* siRNA for 24 h and treated with CSE for an additional 24 h. *n* = 3 per group. Data are presented as means ± SEMs. ^*^ *p* < 0.05; ^**^ *p* < 0.01; ^***^ *p* < 0.001.

To directly explore the functional role of TALDO1 in CS‐induced mitochondrial regulation, *TALDO1* was silenced in HBE cells using siRNA, with knockdown efficiency confirmed by western blotting and RT‐PCR (Figure S6A,B). As shown in Figure 3C, CSE exposure markedly reduced the expression of TOM20, a mitochondrial quality marker, whereas *TALDO1* knockdown significantly restored TOM20 expression. Flow cytometry analysis further demonstrated that *TALDO1* knockdown reduced CSE‐induced mitochondrial ROS production (Figure 3D) and rescued mitochondrial membrane potential (Figure 3E). Consistent results were obtained by JC‐1 fluorescence imaging (Figure S6C). These results were further corroborated by mitochondrial ROS assessments in primary human airway epithelial cells (Figure S7A,B).

Importantly, TALDO1 silencing also restored cellular metabolic capacity under CSE exposure. CSE significantly decreased OCR (oxygen consumption rate) and PER, as evidenced by reduced basal respiration, spare respiratory capacity, basal glycolysis, and compensatory glycolysis. These abnormalities were reversed by *TALDO1* knockdown (Figure 3F,G), indicating that TALDO1contributes to both mitochondrial respiratory dysfunction and impaired glycolytic capacity induced by CS.

Collectively, these results suggest that CS exposure induces coordinated mitochondrial and metabolic abnormalities in airway epithelial cells, and identify TALDO1 as an important regulator of mitochondrial and metabolic dysfunction associated with CS exposure.

### 2.4 *TALDO1* Promotes CSE‐Induced Pro‐Inflammatory and Mucous Responses Through Aggravation of Mitochondrial Dysfunction

In parallel with metabolic alterations, CSE exposure significantly upregulated the expression of inflammatory mediators, including IL‐6, IL‐8, CCL2, and TGF‐β1, as well as the mucin gene *MUC5AC*, with the strongest induction observed at 24 h (Figure 4A,B, Figure S5H). However, *TALDO1* knockdown markedly attenuated this induction (Figure 4A,B). To determine whether mitochondrial oxidative stress contributes to TALDO1‐associated inflammatory and mucus‐related responses, we pretreated cells with Mito‐TEMPO (MT), a mitochondria‐targeted antioxidant that scavenges mtROS. As expected, MT pretreatment effectively suppressed CSE‐induced mtROS accumulation (Figure 4C), confirming its functional efficacy. Notably, MT pretreatment did not alter TALDO1 expression under CSE exposure (Figure 4D,E), suggesting that the protective effects of MT were not mediated through suppression of *TALDO1* expression. Importantly, in *TALDO1*‐silenced cells, pretreatment with MT further reduced CSE‐induced inflammatory mediators and MUC5AC expression (Figure 4F,G), indicating an additional protective effect of mtROS scavenging. Taken together, these findings indicate that *TALDO1* contributes to CSE‐induced pro‐inflammatory and mucus‐related responses, and support an important role for mitochondrial oxidative stress in this process. The additional effect of MT following *TALDO1* knockdown further suggests that mitochondrial dysfunction may partially, rather than exclusively, mediate TALDO1‐associated epithelial responses to CS.

**FIGURE 4:**
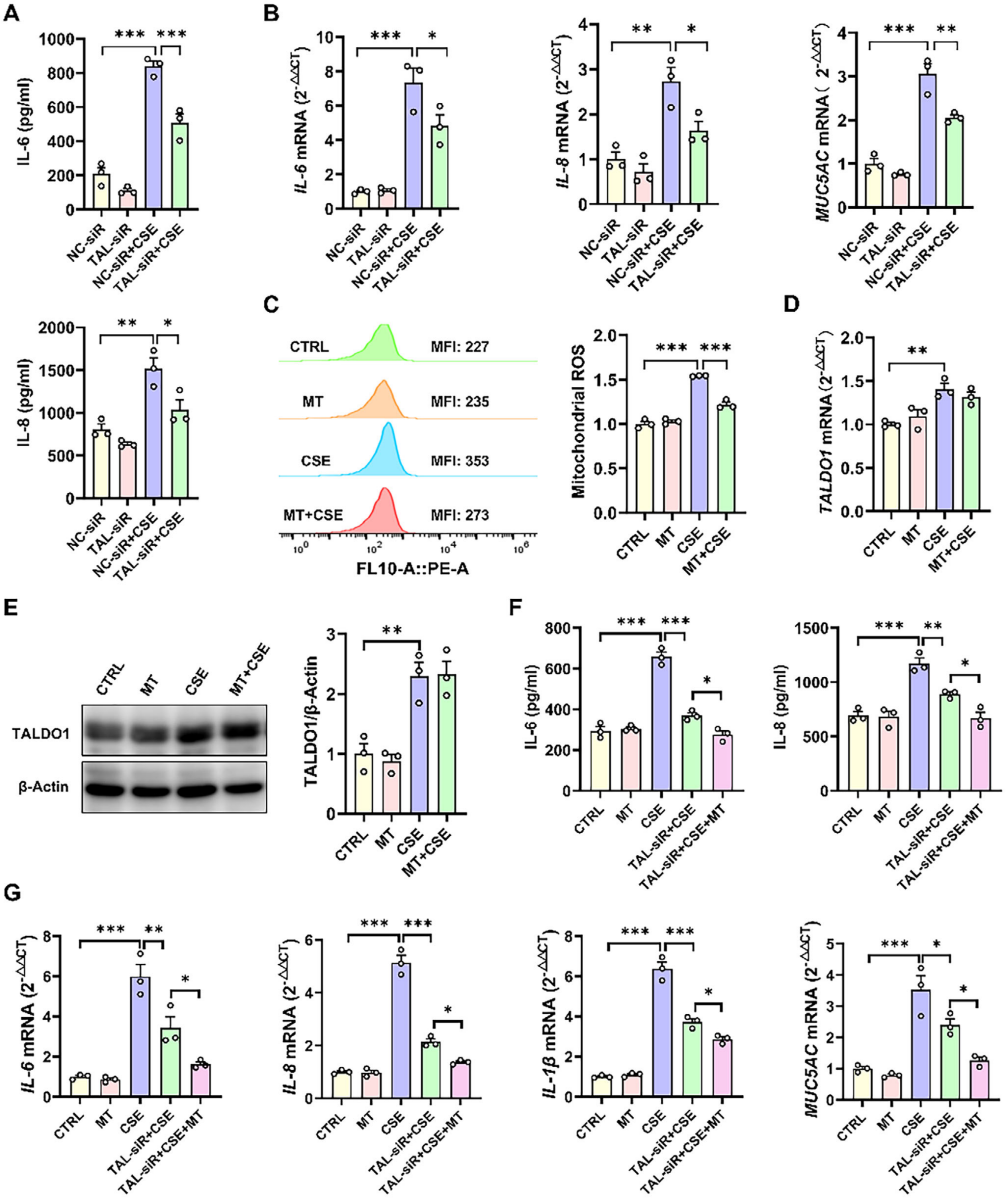
TALDO1 promotes CSE‐induced pro‐inflammatory and mucous responses by aggravating mitochondrial dysfunction. (A, B) HBE cells were transfected with *TALDO1* siRNA for 24 h and treated with CSE for an additional 24 h; then cytokines (IL‐6 and IL‐8) in the supernatant were measured by ELISA (A), and inflammatory and mucus‐related genes (IL‐6, IL‐8, and Muc5ac) were detected by RT‐PCR (B). (C–E) HBE cells were pretreated with 10 µM Mito‐TEMPO (MT) for 2 h before CSE exposure, then Mitochondrial ROS were measured by flow cytometry (C) and TALDO1 expression was measured by RT‐PCR and Western blot, respectively (D, E). (F, G) HBE cells were transfected with *TALDO1* siRNA for 24 h, pretreated with 10 µM MT for 2 h, and subsequently exposed to CSE for an additional 24 h; IL‐6 and IL‐8 expressions in cell supernatant (F), and mRNA levels (G) were analyzed by ELISA and RT‐PCR. *n* = 3 per group. Data are presented as means ± SEMs. ^*^ *p* < 0.05; ^**^ *p* < 0.01; ^***^ *p* < 0.001.

### 2.5 *TALDO1* Promotes L‐Kynurenine (LKU) Accumulation Associated With Mitochondrial Function

To investigate how *TALDO1* influences mitochondrial function, we conducted untargeted metabolomic profiling of HBE cells exposed to CSE, with or without *TALDO1*, to identify metabolites regulated by *TALDO1*. As shown in Figure 5A, L‐kynurenine (LKU) levels were markedly increased upon CSE stimulation; however, this elevation was significantly attenuated following *TALDO1* knockdown, indicating that TALDO1 contributes to CS‐induced LKU accumulation. These findings were further confirmed by LC‐MS/MS, which showed consistent results (Figure 5B). To validate the findings of prior metabolomics studies, we conducted proteomic analysis on lung tissues. The KEGG enrichment analysis revealed that *Taldo1* ^fl/fl^ Cre mice exposed to CS demonstrated significant reprogramming of the lung proteome in comparison to *Taldo1* ^fl/fl^ mice. The pathway with the most pronounced enrichment pertained to amino acid and cofactor metabolism. Notably, the “Tryptophan metabolism” pathway exhibited substantial enrichment, indicating TALDO1 deficiency was associated with altered abundance of proteins involved in this pathway (Figure 5C). We next measured LKU levels in lung tissue and serum from our mouse model. In line with the in vitro observations, CS exposure markedly increased LKU levels in both lung tissues and serum compared with air controls, whereas *Taldo1* airway epithelial‐specific deficiency significantly reduced LKU accumulation in response to CS exposure (Figure 5D), as well as in *Taldo1* ^−/−^ mice (Figure S8). To explore the functional role of LKU in mitochondrial regulation, HBE cells were treated with 50 µM LKU for 24 h. Exogenous LKU markedly induced mtROS production (Figure 5E), supporting a direct effect of LKU on mitochondrial redox homeostasis. Furthermore, pretreatment with Epacadostat (Epa), an IDO1 inhibitor that blocks LKU synthesis, significantly reduced CS‐induced mtROS accumulation in HBE cells (Figure 5F), consistent with a contribution of endogenous kynurenine‐pathway activation to CS‐induced mitochondrial oxidative stress. Collectively, these results identify LKU as a TAOLD1‐associated metabolite that accumulates following CS exposure and contributes to mitochondrial damage. The concordant metabolomic, proteomic, and in vivo data support a model in which TALDO1 may promote remodeling of tryptophan–kynurenine metabolism, thereby contributing to mitochondrial dysfunction under CS exposure.

**FIGURE 5:**
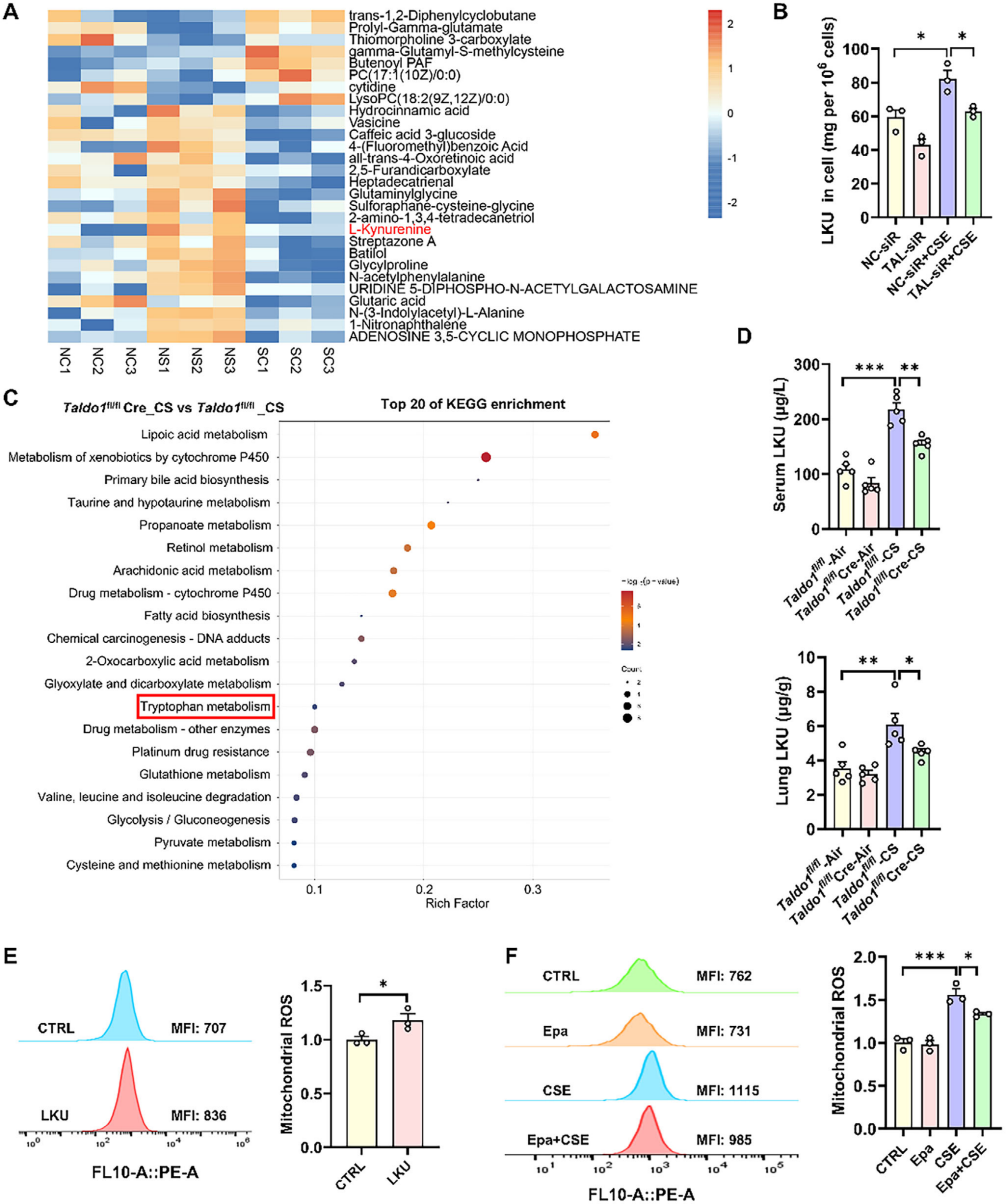
TALDO1 regulates the accumulation of L‐kynurenine (LKU) and its subsequent impact on mitochondrial function. (A) Heatmap of differential metabolites from untargeted metabolomics in HBE cells transfected with *TALDO1* siRNA for 24 h and treated with CSE for an additional 24 h (NC indicates NC‐siR, NS indicates NC‐siR+CSE, and SC indicates TAL‐siR+CSE). (B) LKU levels in HBE cells. (C) KEGG pathway enrichment analysis of differentially expressed proteins in *Taldo1* ^fl/fl^Cre‐CS vs *Taldo1* ^fl/fl^‐CS groups. (D) LKU levels in lung tissues and serum from mice were measured using LC‐MS/MS. (E) mtROS levels in HBE cells treated with 50 µM LKU for 24 h were analyzed by flow cytometry. (F) mtROS levels in HBE cells pretreated with 5 µM Epacadostat (Epa) for 2 h before CSE stimulation were measured by flow cytometry. *n* = 3–5 per group. Data are presented as means ± SEMs. ^*^ *p* < 0.05; ^**^ *p* < 0.01; ^***^ *p* < 0.001.

### 2.6 L‐Kynurenine Serves as the Principal Downstream Effector in the Mediation of Mitochondrial Damage Induced by *TALDO1*

To ascertain whether the accumulation of LKU mediates TALDO1‐induced mitochondrial damage, we conducted a pivotal metabolite replenishment experiment. The findings revealed that TALDO1 knockdown mitigated CSE‐induced mitochondrial dysfunction, corroborating previous observations. Notably, the supplementation of exogenous LKU in TALDO1‐knockdown cells negated this protective effect, as demonstrated by increased mitochondrial reactive oxygen species (mtROS) levels and a reduction in mitochondrial membrane potential (Figure 6A,B). Moreover, LKU supplementation significantly compromised mitochondrial respiration, including basal respiration and spare respiratory capacity, as well as glycolytic function, encompassing both basal glycolysis and compensatory glycolysis (Figure 6C,D). These results provide direct experimental evidence supporting the causal relationship wherein TALDO1 promotes mitochondrial dysfunction through the induction of LKU accumulation, thereby identifying LKU as the principal pathogenic metabolite downstream of TALDO1.

**FIGURE 6:**
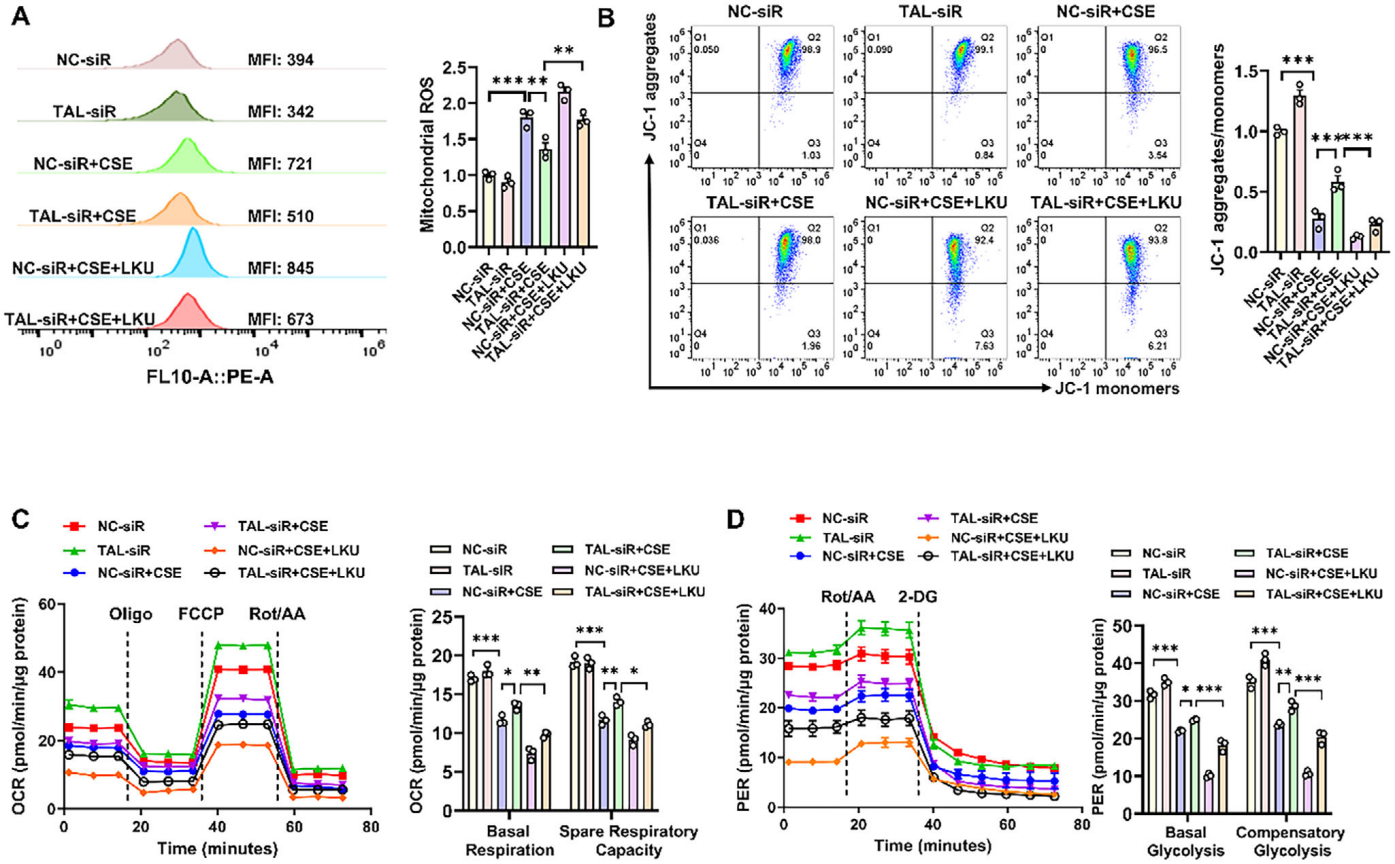
L‐kynurenine functions as the primary downstream effector in mediating mitochondrial damage induced by *TALDO1*. (A–D) mtROS production (A), mitochondrial membrane potential (B), OCR (C), and PER (D) were analyzed in HBE cells with LKU replenishment after *TALDO1* knockdown and CSE stimulation. *n* = 3 per group. Data are presented as means ± SEMs. ^*^ *p* < 0.05; ^**^ *p* < 0.01; ^***^ *p* < 0.001.

### 2.7 Epa Alleviates CS‐Induced Lung Damage, Inflammation, and Mitochondrial Dysfunction in Mouse Model

To evaluate the therapeutic potential of targeting kynurenine metabolism in COPD, CS‐exposed mice were treated with Epa, a selective IDO1 inhibitor that suppresses kynurenine synthesis, for two months following four months of CS exposure, as illustrated in Figure 7A. Consistent with our previous findings, CS exposure markedly reduced body weight, impaired pulmonary function, increased inflammatory cell accumulation in BAL fluid, elevated BAL levels of IL‐6 and CCL2, and induced emphysematous and airway‐remodeling changes (Figure 7B–G). Notably, Epa treatment did not significantly alter body weight (Figure 7B), but it yielded substantial protective effects against CS‐induced pulmonary pathology. Pulmonary function analysis showed that Epa reduced CS‐induced lung hyperinflation, as indicated by decreased FRC, and alleviated airflow limitation, as reflected by increased FEV50/FVC (Figure 7C). Epa administration also significantly attenuated CS‐driven airway inflammation, with reduced total inflammatory cell infiltration and a lower proportion of neutrophils in BAL fluid compared with vehicle‐treated CS‐exposed mice (Figure 7D). These cellular changes were accompanied by decreased BAL levels of IL‐6 and CCL2 (Figure 7E).

**FIGURE 7:**
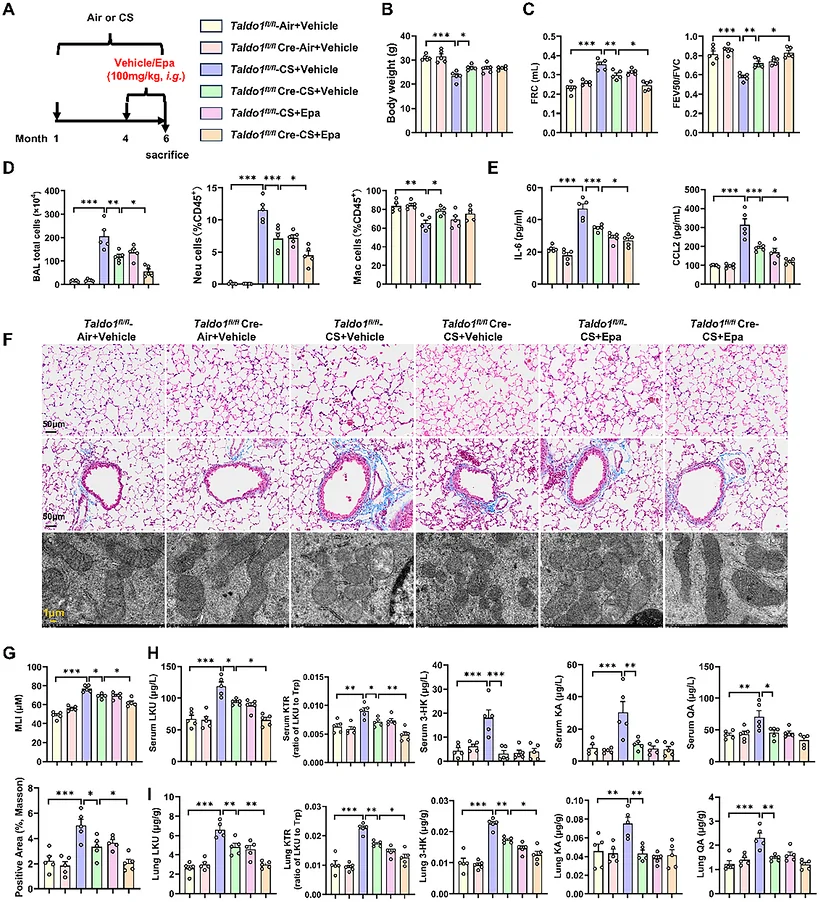
Epa alleviates CS‐induced lung dysfunction and structural damage in a mouse model. (A) Schematic illustration of the experimental protocol for CS exposure and Epa administration. (B) Body weight analysis. (C) Pulmonary function detection. (D) BAL fluid analysis of total cell counts and differential cells by flow cytometry. (E) ELISA measurement of cytokines in BAL fluid. (F) Representative H&E staining (upper panel), Masson's trichrome staining (middle panel), and TEM images (lower panel) of lung tissues. (G) Quantitative analysis of MLI and peribronchial collagen deposition. (H, I) LC‐MS/MS analysis of tryptophan‐kynurenine pathway metabolites in serum (H) and lung tissues (I). *n* = 5 per group. Data are presented as means ± SEMs. ^*^ *p* < 0.05; ^**^ *p* < 0.01; ^***^ *p* < 0.001.

Histopathological analysis further showed that Epa ameliorated CS‐induced structural lung injury. H&E staining demonstrated reduced alveolar enlargement, as demonstrated by a reduced MLI, whereas Masson's trichrome staining showed reduced peribronchial collagen deposition, indicating attenuation of airway remodeling (Figure 7F,G, Figure S9, upper panel). Consistently, transmission electron microscopy revealed improved mitochondrial ultrastructure after Epa intervention, characterized by more clearly defined cristae and reduced mitochondrial matrix swelling (Figure 7F).

To determine whether these phenotypic improvements were associated with modulation of the kynurenine metabolic axis, targeted LC‐MS/MS analysis was performed in serum and lung tissues. Epa treatment significantly reduced LKU levels and the LKU/Trp ratio (KTR) in both compartments (Figure 7H,I). In contrast, levels of several downstream kynurenine pathway metabolites, including kynurenic acid (KA), quinolinic acid (QA), and 3‐hydroxykynurenine (3‐HK), remained largely unchanged (Figure 7H,I). These findings indicate that Epa preferentially reduced LKU accumulation under the present experimental conditions. Collectively, these results demonstrate that pharmacological inhibition of IDO1 attenuates CS‐induced lung dysfunction, inflammation, structural damage, and mitochondrial injury, in parallel with reduced LKU accumulation. Together with the preceding genetic and metabolic data, these findings further support the involvement of altered TALDO1‐associated kynurenine metabolism in CS‐induced lung injury.

## 3 Discussion

This study demonstrates that CS exposure markedly upregulates TALDO1 expression in airway epithelial cells and identifies TALDO1 as a previously unrecognized metabolic regulator involved in COPD pathogenesis. Mechanistically, TALDO1 upregulation promotes the accumulation of L‐kynurenine, thereby contributing to CS‐induced mitochondrial dysfunction. Genetic loss‐of ‐function experiments in vivo and in vitro support a functional relationship among TALDO1 expression, L‐kynurenine accumulation, and mitochondrial injury, indicating that TALDO1 acts upstream of kynurenine‐associated epithelial damage. Importantly, pharmacological inhibition of IDO1 with Epa, which suppresses kynurenine synthesis, significantly attenuates CS‐induced mitochondrial dysfunction, inflammatory responses, and COPD‐like pathological changes in mice. Together, these findings position TALDO1 as an important regulator linking CS exposure to airway epithelial metabolic reprogramming, mitochondrial dysfunction, inflammation, and COPD progression and suggest targeting kynurenine metabolism may represent a potential therapeutic strategy.

Our findings align with the growing body of evidence supporting the role of metabolic reprogramming in COPD [32, 33, 34]. CS induces a wide range of metabolic adaptations, and the upregulation of TALDO1, a key enzyme of the PPP, may serve as a compensatory response to oxidative damage. TALDO1 is a key enzyme of the non‐oxidative PPP, catalyzing the reversible conversion of sedoheptulose‐7‐phosphate and glyceraldehyde‐3‐phosphate to erythrose‐4‐phosphate and fructose‐6‐phosphate [35]. This reaction contributes to the balance between pentose phosphate metabolism and the recycling of carbon skeletons into glycolysis. However, under sustained CS exposure, persistent TALDO1 upregulation was associated with detrimental metabolic consequences. TALDO1 upregulation occurred despite reduced mitochondrial respiratory capacity and glycolytic capacity, highlighting the distinction between enzyme abundance and net metabolic flux. Consistently, TALDO1 knockdown partially restored basal respiration, spare respiratory capacity, basal glycolysis, and compensatory glycolysis following CSE exposure. These findings suggest that sustained TALDO1 dysregulation is associated with impaired metabolic flexibility rather than a simple increase in the PPP‐derived carbon flux into glycolysis. Direct metabolic flux analysis will be required to determine how TALDO1 quantitatively alters carbon partitioning between the PPP and glycolysis under CS exposure.

Redox imbalance is closely linked to mitochondrial dysfunction, as excessive ROS can impair mitochondrial membrane potential and respiratory function, while damaged mitochondria can further amplify oxidative stress and inflammatory signaling. Consistent with this concept, the present findings indicate that TALDO1 is associated with CS‐induced mitochondrial dysfunction and inflammatory responses, whereas TALDO1 silencing attenuates these abnormalities. Mito‐TEMPO treatment further reduced inflammatory cytokine production and *Muc5ac* expression in *TALDO1*‐silenced cells, suggesting that mitochondrial oxidative stress contributes, at least in part, to TALDO1‐associated inflammatory and mucus‐related responses. Thus, TALDO1‐associated metabolic reprogramming may couple altered cellular metabolism to mitochondrial injury and sustained epithelial inflammation. In this context, *TALDO1*‐mediated metabolic reprogramming does not merely accompany cellular stress but may actively shape the inflammatory and secretory phenotype of airway epithelial cells. Our finding that *TALDO1* knockdown attenuated both CS‐induced inflammation and mitochondrial dysfunction further supports a close interaction between metabolic reprogramming and inflammatory signaling. Beyond COPD, TALDO1‐dependent metabolic rewiring has been implicated in other pathological contexts, including cancer cell proliferation and immune cell activation [36, 37], suggesting that the metabolic‐inflammatory relationship identified here may represent a broader mechanism linking cellular metabolism to tissue inflammation and remodeling.

A particularly important finding is the relationship between TALDO1 and kynurenine metabolism. Untargeted metabolomics identified L‐kynurenine as a metabolite markedly increased following CSE exposure and significantly reduced by TALDO1 knockdown. This relationship was further supported by targeted LC–MS/MS, proteomic enrichment of tryptophan‐metabolism pathways, and reduced L‐kynurenine concentrations in lung tissue and serum following TALDO1 deficiency. Exogenous L‐kynurenine induced marked mitochondrial oxidative stress. Whereas genetic suppression of TALDO1 reduced L‐kynurenine accumulation and mitochondrial injury. Moreover, supplementation with L‐kynurenine partially restored the mitochondrial abnormalities attenuated by TALDO1 suppression, supporting kynurenine as an important downstream effector of TALDO1‐associated metabolic remodeling. Nevertheless, the precise biochemical mechanism linking TALDO1 to kynurenine metabolism remains to be established.

Kynurenine is increasingly recognized as an immunometabolic mediator with context‐dependent biological effects in lung diseases. Recent studies have suggested that kynurenine may exert anti‐fibrotic effects through the AHR‐PTEN axis in pulmonary fibrosis, although such protective effects may be modified by the pathological microenvironment [38]. Kynurenine also participates in immune regulation by shaping the Th17/Treg immune balance in asthma and infectious diseases [39, 40, 41]. In COPD, increased plasma kynurenine levels and an elevated kynurenine‐to‐tryptophan ratio have been reported in patients with mild‐to‐moderate disease [42, 43]; however, whether these metabolic alterations contribute to disease progression or merely reflect systemic inflammation has remained unclear. The present study provides functional evidence that kynurenine accumulation is not simply a bystander metabolic change in COPD, but contributes to CS‐induced epithelial injury. A central effect identified here is the impact of L‐kynurenine on mitochondrial redox homeostasis. L‐kynurenine treatment markedly increases mtROS generation in airway epithelial cells, linking kynurenine accumulation to mitochondrial oxidative stress. These findings are consistent with observations in other disease models showing that kynurenine‐pathway metabolites can compromise mitochondrial hemeostasis [44, 45, 46], but extend this concept to COPD by identifying TALDO1 as an upstream metabolic regulator associated with kynurenine accumulation.

Importantly, this study further demonstrates the therapeutic relevance of this pathway. Treatment with Epa, a selective IDO1 inhibitor that suppresses kynurenine synthesis, significantly attenuated CS‐induced mitochondrial dysfunction, lung inflammation, structural injury, and COPD‐like disease progression in mice. These results indicate that altered TALDO1‐associated kynurenine metabolism is not only mechanistically relevant, but may also be pharmacologically modifiable. However, because Epa acts directly on IDO1 rather than TALDO1 or kynurenine itself, these results should not be interpreted as definitive evidence of a strictly linear TALDO1–IDO1–kynurenine pathway. Instead, they support the functional importance of kynurenine‐pathway activity downstream of TALDO1‐associated metabolic remodeling. Therefore, targeting kynurenine metabolism may represent a promising translational strategy to mitigate CS‐induced epithelial injury and slow COPD progression.

Beyond mitochondrial dysfunction and kynurenine metabolism, TALDO1 dysregulation may also influence epithelial homeostasis. The non‐oxidative PPP contributes to the recycling of carbon substrates involved in nucleotide metabolism [27, 31, 47]. Under physiological conditions, PPP metabolism is closely linked to the nucleotide pool homeostasis required for cell proliferation, DNA repair, and genome maintenance [48, 49]. Meanwhile, COPD airway epithelium is characterized by accumulated DNA damage, cellular senescence, and impaired barrier function [50, 51, 52]. Abnormal upregulation of TALDO1 may also compromise genomic stability and epithelial regenerative capacity by disturbing nucleotide metabolism. It is possible that TALDO1‐associated metabolic reprogramming disrupts nucleotide pool homeostasis, thereby impairing DNA repair and regeneration processes. However, this possibility was not directly examined in the present study and requires further investigation.

Several limitations should be acknowledged. The precise metabolic mechanism linking TALDO1 to the tryptophan–kynurenine pathway remains unclear and warrants metabolic flux analysis and direct assessment of kynurenine‐pathway enzymes. The potential contributions of downstream kynurenine metabolites require further evaluation. In addition, all animal experiments were conducted in male mice, and the human cohort was not powered for sex‐stratified analyses; therefore, possible sex‐dependent effects of TALDO1 remain unresolved. Finally, although no overt hepatic injury was observed following Epa treatment (Figure S9, lower panel), its systemic effects on other organs and long‐term safety remain to be determined.

In summary, our findings identify TALDO1‐associated accumulation of L‐kynurenine as a previously unrecognized metabolic mechanism contributing to CS‐induced mitochondrial dysfunction. This discovery provides a new metabolic perspective on COPD pathogenesis and suggests that targeting the TALDO1‐kynurenine axis may be a promising strategy for slowing disease progression. Our work not only deepens the understanding of the metabolic etiology of COPD, but also uncovers potential therapeutic targets. Although directly targeting TALDO1, an essential metabolic enzyme, requires careful safety evaluation, modulation of downstream kynurenine metabolism may offer a more feasible and specific therapeutic approach. Future studies should determine whether TALDO1‐associated metabolic signatures can define clinically relevant COPD subtypes and whether such patients may preferentially benefit from metabolism‐directed interventions.

## 4 Experimental Section

### 4.1 Human Specimens

Cells were collected by brushing from healthy controls and COPD patients. All human specimens were obtained from the First Affiliated Hospital of Guangzhou Medical University, with informed consent acquired from all participants. The study was approved by the Medical Ethics Committee of Guangzhou National Laboratory (Reference number: 2023‐0009). Clinical data are summarized in Table S1.

### 4.2 Animals

The airway epithelial‐specific *Taldo1* knockout mice were generated by cross‐breeding with *Taldo1^flox/flox^* and *Scgb1a1*‐IRES‐Cre transgenic mice as shown in Figure S2A. *Taldo1^flox/flox^* mice were produced utilizing the CRISPR/Cas‐mediated genome engineering system by Cyagen Biosciences Inc. (Suzhou, China). Specifically, two loxP sequences were introduced into the introns surrounding exons 2–4 of *Taldo1* as depicted in Figure S2A. The *Scgb1a1*‐IRES‐Cre transgenic mice were purchased from the Shanghai Model Organism Center (Shanghai, China). *Taldo1^±^* mice on the C57BL/6J background were purchased from Cyagen Biosciences Inc. (Suzhou, China), with wild‐type (WT) littermates as controls. Mice, aged of 6–8 weeks, were used in age‐ and sex‐matched groups. All mice were housed in a specific pathogen‐free animal facility under controlled temperature and humidity, with free access to food and water. Experimental procedures were approved by the Ethics Committee of Guangzhou National Laboratory (GZLAB‐AUCP‐2023‐02‐A04) and followed the Guide for the Care and Use of Laboratory Animals.

### 4.3 CS‐Induced Mouse Model

The chronic CS‐induced model was established as previously described [53]. Briefly, mice were exposed to CS from Hongmei cigarettes (tar: 13 mg, nicotine:0.8 mg per cigarette) using a whole‐body passive smoking system (Liuwei Hardware & Electromechanical Co., Ltd., Guangzhou, China). After a two‐week adaptation, mice were exposed to smoke for 4 h daily, a total of 48 cigarettes per day for six months. Age‐ and sex‐matched controls were housed in a clean‐air chamber under identical conditions. In separate experiments, mice were treated with vehicle or Epa, an inhibitor of kynurenine synthesis, via intragastric administration (100 mg/kg) for two months following four months of CS exposure.

### 4.4 Pulmonary Function Detection

Mice were anesthetized with 2% Avertin (1.5 mL/kg) via intraperitoneal injection, followed by endotracheal intubation. The mice were then placed in a DSI pulmonary function test system (Wilmington, NC, USA) to assess their pulmonary function according to the manufacturer's instructions. Parameters were analyzed, including forced vital capacity (FVC), chord compliance (Cchord) at 0–10 cm H_2_O, and forced expiratory volume (FEV) at 50 ms (FEV50) or 100 ms (FEV100).

### 4.5 ATP Production Measurement

The culture medium was removed, and 100 µL of lysis buffer was added to each well of a 12‐well plate. After lysis, samples were centrifuged at 12 000 ×g for 5 min at 4°C, and the supernatant was collected for ATP measurement using the ATP Assay Kit (Beyotime Biotechnology, # S0027) according to the manufacturer's instructions.

### 4.6 Histopathology, Immunohistochemistry, and Immunofluorescence

Mouse lungs were fixed with 4% formalin and sectioned at 4 µm. Sections were deparaffinized, dehydrated, and stained with hematoxylin and eosin (H&E), or subjected to immunohistochemistry or immunofluorescence as previously reported [54].

### 4.7 Mitochondrial Ultrastructure

Fresh lung tissues were collected, cut into 1‐mm^3^ pieces, fixed, and dehydrated at room temperature. The samples were then infiltrated and embedded in resin and polymerized. Embedded blocks were trimmed and sectioned into ultrathin slices using an ultramicrotome. Sections were stained with electron‐dense stains and dried overnight. Ultrathin sections were examined using a transmission electron microscope (TEM, HITACHI, HT7800).

### 4.8 Cell Culture and Treatment

Human bronchial epithelial (HBE) cells were cultured in RPMI‐1640 medium with 10% fetal bovine serum and 1% penicillin/streptomycin in a 37°C incubator with 5% CO_2_. Cells were treated with CSE at the indicated concentrations and durations. For knockdown experiments, HBE cells were transfected with *TALDO1‐*targeting siRNA or a negative control (GenePharma, Shanghai, China) using Lipofectamine 3000. In intervention experiments, HBE cells were pretreated with 5 µM Epa (MedChemExpress, # HY‐15689) for 2 h before CSE stimulation or treated with 50 µM LKU (MedChemExpress, # HY‐104026) for 24 h, respectively.

### 4.9 Seahorse

Oxygen consumption rate (OCR) and proton efflux rate (PER) were measured using the XF Cell Mito Stress Test kit (Agilent Technologies, # 103015–100) and XF Glycolytic Rate Assay Kit (Agilent Technologies, # 103344‐100) following the manufacturer's instructions. Briefly, HBE cells were seeded at 10 000 cells per well in XF96 plates. For the mitochondrial stress test, oligomycin (1.5 µM), carbonyl cyanide‐4‐(trifluoromethoxy) phenylhydrazone (FCCP, 1 µM), and a mixture of antimycin A and rotenone (Rot/AA, 0.5 µM) were sequentially added to assess mitochondrial respiration, including basal respiration and spare respiratory capacity. For the glycolytic rate assay, a mixture of Rot/AA (0.5 µM) and 2‐deoxy‐D‐glucose (50 mM) was added to measure basal glycolysis and compensatory glycolysis via PER.

### 4.10 mtROS Measurement

Levels of mtROS were assessed using the MitoSOX Red probe (Thermo Fisher Scientific, # M22425). Cells were cultured to 80% confluence in 12‐well plates, harvested and centrifuged at 1000 rpm for 5 min. After removing the supernatant, cells were incubated with 5 µM MitoSOX for 15 min at 37°C in the dark. mtROS levels were then analyzed by flow cytometry (Beckman, USA).

### 4.11 Mitochondrial Membrane Potential Assay

Mitochondrial membrane potential was measured using the JC‐1 assay kit (Beyotime Biotechnology, # C2006). Cells were cultured to 80% confluence, then incubated with JC‐1 dye for 15 min at 37°C in the dark. After staining, cells were imaged with a confocal microscope or analyzed by flow cytometry (Beckman, USA) to quantify JC‐1 fluorescence shifts.

### 4.12 L‐Kynurenine Measurement

For sample preparation, 200 µL of MeOH/ACN/H_2_O (v: v: v, 2:2:1) was added to 50 µL of serum or lung tissue homogenate supernatant. The mixture was vortexed for 30 s, sonicated in a 4°C water bath for 10 min, and incubated at −20°C for 1 h to precipitate proteins. After centrifugation at 16 500 ×g for 15 min at 4°C, 200 µL of the supernatant was collected for Liquid Chromatography‐tandem Mass Spectrometry (LC‐MS/MS) analysis.

### 4.13 Quantitative Real‐Time PCR (qRT‐PCR)

Total RNA was isolated from cells and mouse lung tissues using TRIzol universal reagent (Takara, #9109) according to the manufacturer's instructions. cDNAs were synthesized with the PrimeScript RT Reagent Kit with gDNA Eraser (Takara, #RR037A). The cDNA was then subjected to real‐time PCR (Bio‐Rad) with GAPDH as an internal control. Primer details are provided in Table S3.

### 4.14 Western Blotting

Proteins were extracted from samples with RIPA buffer (Thermo Fisher Scientific, #89901), and concentrations were determined. Equal amounts of protein were denatured, separated by SDS‐PAGE, and transferred onto PVDF membranes. Membranes were blocked with 5% fat‐free milk and incubated with primary and corresponding secondary antibodies listed in Table S4. Protein expression was visualized using an automated chemiluminescence imaging system. Original uncropped images of all Western blot membranes are provided in Figure S10.

### 4.15 Statistical Analysis

Data were analyzed using GraphPad Prism 10.2.0 and presented as Means ± S.E.M. Differences among groups were assessed with one‐way ANOVA followed by the Student‐Newman‐Keuls (SNK) test. The unpaired Student's t‐test was applied for comparisons between two groups. Statistical significance was set at *p* < 0.05, with ^*^ *p* < 0.05, ^**^ *p* < 0.01, and ^***^ *p* < 0.001.

## Author Contributions

W.W., M.W., and Y.Y. carried out the majority of the experiments, analyzed and interpreted the data, and contributed equally to the paper. Z.C. and J.Y. participated in the animal studies. Y.C. performed the bioinformatics analysis. F.Z., X. J., H.L., and W. C. provided technical advice. Y. Z., L.Z., and P.R. supervised the research and provided the funding. W.W. and T.W. wrote the initial manuscript and revised the manuscript. All authors have read and approved the final version of the manuscript.

## Funding

This work was supported by the Major Project of Guangzhou National Laboratory (GZNL2025C02006), the Foundation of Guangzhou National Laboratory (SRPG22‐016, SRPG22‐018, and GZNL2023A02001), the National Natural Science Foundation of China (81970045, 82270043, and 82400036), and the Young Scientists Program of Guangzhou National Laboratory (QNPG24‐19).

## Conflicts of Interest

The authors declare no conflicts of interest.

## Supporting information




**Supporting File**: advs78795‐sup‐0001‐SuppMat.docx

## Data Availability

All data necessary to understand and evaluate the study's conclusions are provided in the article and Supplementary Information. Additional data are available from the corresponding author upon request.
