# Supplementary material for: Targeting Kynurenine Synthesis Attenuated Cigarette Smoke‐Induced COPD‐Like Lung Injury Driven by TALDO1‐Mediated Metabolic Reprogramming
Source: Adv Sci (Weinh). 2026 Nov 11:e78795. Online ahead of print. doi: 10.1002/advs.78795 (PMC13753984; doi:10.1002/advs.78795)
Supplement: Supplementary file 1 [file ADVS-9999-e78795-s001.docx]

Targeting Kynurenine Synthesis Attenuated Cigarette Smoke–Induced COPD-like Lung Injury Driven by TALDO1-Mediated Metabolic Reprogramming

Wenjing Wang^1,2#^, Mindan Wu^1,2#^, Yuanlan Yang^3#^, Zemin Chen^1,2^, Jiawei Yan^3^, Xuan Jin^2^, Yachao Cui^1,2^, Feng Zhang^1,2^, Haiqing Li^1^, Wensheng Chen^2^, Yumin Zhou^1,2*^, Leqing Zhu^2*^, Wei Tu^2*^, Pixin Ran^1,2*^

1. State Key Laboratory of Respiratory Diseases, National Clinical Research Center for Respiratory Diseases, Guangzhou Institute of Respiratory Health, the First Affiliated Hospital of Guangzhou Medical University. Guangzhou 510005, Guangdong, China.
2. Guangzhou National Laboratory. No.9 XingDaoHuanBei Road, Guangzhou International BioIsland, Guangzhou 510005, Guangdong, China.
3. GMU-GIBH Joint School of Life Sciences, Guangzhou Medical University, Guangzhou 511436, China.

#These authors contributed equally to this work.

***Corresponding author:** Pixin Ran, Email: [pxran@gzhmu.edu.cn](mailto:pxran@gzhmu.edu.cn); Wei Tu, Email: [tuwei88@yeah.net](mailto:tuwei88@yeah.net); Leqing Zhu, Email: [zhuleqing1@163.com](mailto:zhuleqing1@163.com); Yumin Zhou, Email: [zhouyumin410@126.com](mailto:zhouyumin410@126.com). State Key Laboratory of Respiratory Diseases, National Clinical Research Center for Respiratory Diseases, Guangzhou Institute of Respiratory Health, the First Affiliated Hospital of Guangzhou Medical University. Guangzhou 510005, Guangdong, China.

**Materials and methods**

**Bronchoalveolar lavage (BAL) analysis**

Freshly collected BAL fluid was centrifuged 800×g for 5 minutes at 4 ℃, and the supernatant was stored at -80 ℃ for further analysis. The pellets were used for total cell count and differential cell analysis. Briefly, cells were blocked and incubated with antibodies listed in Supplemental Table S2. After red blood cell lysis, cells were resuspended, mixed with counting microspheres, and analyzed by flow cytometry as previously reported[1].

**Enzyme-linked immunosorbent assay (ELISA)**

Cell-free BALF supernatant were subjected to ELISA for IL-6, IL-1β, CCL2 and TNF-α measurement with ELISA kits (Thermo Fisher Scientific, USA) according to manufacturer’s instructions.

**Untargeted Metabolomics**

For untargeted metabolomics analysis, raw data files were converted to mzXML format using ProteoWizard, followed by peak extraction, quantification, and alignment across samples using XCMS based on retention time and mass-to-charge ratio. Metabolite identification was performed by matching against the Novogene in-house untargeted metabolomics database with a mass tolerance of 10 ppm and consideration of adduct ions, with data processing conducted on a Linux system (CentOS 6.6) using R and Python. The preprocessing pipeline included: removal of metabolites with >50% missing values (excluding QC samples) followed by KNN imputation; background subtraction by excluding metabolites with higher peak areas in Blank than in QC1 samples; QC normalization using the formula: sample intensity/(sum of sample intensities/sum of QC1 intensities); removal of metabolites with coefficient of variation exceeding 30% in QC samples; and deduplication based on InChIKey or English name, retaining the compound with the lower confidence level or higher identification score. Metabolites were annotated using the KEGG, HMDB, and LIPIDMaps databases. For multivariate analysis, PCA and PLS-DA were performed using metaX to derive VIP values, while univariate analysis used Student's t-test to calculate P-values and fold changes (FC), with differentially abundant metabolites defined as VIP > 1, P < 0.05, and FC > 2 or FC ≤ 0.5. Volcano plots were generated using ggplot2 integrating VIP, log2(FC), and −log10(P-value); heatmaps were generated using pheatmap with z-score normalization; Pearson correlation analysis among differential metabolites was performed using the R function cor () with significance assessed by cor.mtest() (P < 0.05), and correlation plots were generated using corrplot.

**Tab.S1. Characteristics of the participants for bronchial brush study**

| Groups | Sex | Age (years) | Smoking status | Smoking Index(pack-year) | mMRC score | Clinical diagnosis |
| --- | --- | --- | --- | --- | --- | --- |
| Control 1 | M | 55 | Never smoking | 0 | 0 | Lung opacity |
| Control 2 | F | 62 | Never smoking | 0 | 0 | Lung mass |
| Control 3 | M | 56 | Never smoking | 0 | 0 | Cough |
| COPD 1 | M | 70 | Current smoking | 50 | 0 | COPD,  GOLD 1 |
| COPD 2 | M | 64 | Current smoking | 50 | 0 | COPD, GOLD3 |
| COPD 3 | M | 76 | Current smoking | 60 | 0 | COPD, GOLD1 |
| COPD 4 | M | 65 | Former smoking | 60 | 0 | COPD, GOLD3 |
| COPD 5 | M | 60 | Former smoking | 30 | 0 | COPD, GOLD1 |

**Tab. S2. Antibodies for flow cytometry.**

| Antibody | Clone | Host | Fluorophore | Manufacturer | Product# |
| --- | --- | --- | --- | --- | --- |
| Zombie NIR |  |  | Ex~650nm, Em~785nm | Biolegend | 423105 |
| CD16/32 | S17011E | Rat |  | Biolegend | 156604 |
| CD45 | S18009F | Mouse | PE/Cyanine7 | Biolegend | 157206 |
| CD11c | N418 | Hamster | BV421 | Biolegend | 117330 |
| Siglec-F | S17007L | Rat | PE | Biolegend | 155506 |
| MHC-II | M5/114.15.2 | Rat | ef450 | eBioscience | 48-5321-82 |
| CD3ε | 145-2C11 | Hamster | Percp | Biolegend | 100326 |
| TCR-β | H57-597 | Hamster | APC/Cyanine7 | Biolegend | 109220 |
| CD11b | M1/70 | Rat | FITC | Biolegend | 101206 |
| Ly6G（Gr-1） | RB6-8C5 | Rat | APC | Biolegend | 108412 |

**Tab. S3. Sequences of the primers for real-time PCR.**

| Genes | Sequences | Species |
| --- | --- | --- |
| *Gapdh* | Forward: 5'-AGGTCGGTGTGAACGGATTTG-3' | Mouse |
|  | Reverse: 5'-TGTAGACCATGTAGTTGAGGTCA-3' |  |
| *Taldo1* | Forward: 5'-AAGAAGATTCCAGGCCGTGT-3' | Mouse |
|  | Reverse: 5'-GTCATGTTGCAGTGGATGCC-3' |  |
| *Muc5ac* | Forward: 5'-AATGACTCAATCTGCGTGCCTTCC-3' | Mouse |
|  | Reverse: 5'-CAGGTTAGCGTGGCTTCCTTACAG-3' |  |
| *Cxcl1* | Forward: 5'-ACTCAAGAATGGTCGCGAGG-3' | Mouse |
|  | Reverse: 5'-GTGCCATCAGAGCAGTCTGT-3' |  |
| *Cxcl2* | Forward: 5'-AGGGCGGTCAAAAAGTTTGC-3' | Mouse |
|  | Reverse: 5'-CAGGTACGATCCAGGCTTCC-3' |  |
| *Ccl2* | Forward: 5'-GGCTGGAGAGCTACAAGAGG-3' | Mouse |
|  | Reverse: 5'-GGTCAGCACAGACCTCTCTC-3' |  |
| *Tnf-α* | Forward: 5'-ACTGAACTTCGGGGTGATCG-3' | Mouse |
|  | Reverse: 5'-TGGTGGTTTGTGAGTGTGAGG-3' |  |
| *Mmp12* | Forward: 5'-GAACTTGCAGTCGGAGGGAA-3' | Mouse |
|  | Reverse: 5'-TGCTGGGAACCTTCAGCAAA-3' |  |
| *Mmp13* | Forward: 5'-TTTGAGAACACGGGGAAGA-3' | Mouse |
|  | Reverse: 5'-ACTTTGTTGCCAATTCCAGG-3' |  |
| *IL-1β* | Forward: 5'-GAAATGCCACCTTTTGACAGTG-3' | Mouse |
|  | Reverse: 5'-TGGATGCTCTCATCAGGACAG-3' |  |
| *GAPDH* | Forward: 5'-TGTTGCCATCAATGACCCCTT-3' | Human |
|  | Reverse: 5'-CTCCACGACGTACTCAGCG-3' |  |
| *MUC5AC* | Forward: 5'-CAGCACAACCCCTGTTTCAAA-3' | Human |
|  | Reverse: 5'-GCGCACAGAGGATGACAGT-3' |  |
| *TALDO1* | Forward: 5'-CAGCACAACCCCTGTTTCAAA-3' | Human |
|  | Reverse: 5'-GCGCACAGAGGATGACAGT-3' |  |
| *IL-6* | Forward: 5'-AAGCCAGAGCTGTGCAGATGAGTA-3' | Human |
|  | Reverse: 5'-TGTCCTGCAGCCACTGGTTC-3' |  |
| *IL-8* | Forward: 5'-AACTGAGAGTGATTGAGAGTGG-3' | Human |
|  | Reverse: 5'-ATGAATTCTCAGCCCTCTTCAA-3' |  |
| *CCL2* | Forward: 5'-CTTCTGTGCCTGCTGCTCATA-3' | Human |
|  | Reverse: 5'-CTTTGGGACACTTGCTGCTG-3' |  |
| *TGF-β1* | Forward: 5'-GGCCAGATCCTGTCCAAGC-3' | Human |
|  | Reverse: 5'-GTGGGTTTCCACCATTAGCAC-3' |  |
| *IL-1β* | Forward: 5'-AACCTCTTCGAGGCACAAGG-3' | Human |
|  | Reverse: 5'-AGCCATCATTTCACTGGCGA-3' |  |

**Tab. S4. Antibodies for Western blot and Immunofluorescence.**

| Antibody | Clone | Host | Fluorophore | Manufacturer | Product# |
| --- | --- | --- | --- | --- | --- |
| TALDO1 | H-4 | Mouse |  | Santa Cruz | sc-166230 |
| Tom20 | ARC5002-01 | Rabbit |  | ABclonal | A19403 |
| β-Actin | 8H10D10 | Mouse |  | CST | 3700S |
| HRP-Goat Anti-Mouse Recombinant Secondary Antibody |  | Rabbit |  | Proteintech | RGAR001 |
| HRP-Goat Anti-Mouse Recombinant Secondary Antibody |  | Mouse |  | Proteintech | RGAM001 |
| Goat anti-Mouse IgG1 Cross-Adsorbed Secondary Antibody | Polyclonal | Mouse | Alexa Fluor 594 | Invitrogen | A21125 |
| Goat anti-Rabbit IgG Cross-Adsorbed Secondary Antibody | Polyclonal | Rabbit | Alexa Fluor 488 | Invitrogen | A11008 |

**Figure legends**

**Fig.S1. TALDO1 is upregulated in GSE10006 dataset and CS-induced mouse model. (A)** Volcano plot of differentially expressed genes in GSE10006. **B-C)** TALDO1 expression in mouse lung tissues were measured by Western blot (**B**) and RT-PCR (**C**). Data are presented as means ± SEMs. **p* < 0.05; ***p* < 0.01; ****p* < 0.001.

**Fig.S2. Generation and validation of airway epithelial specific *Taldo1* knockout mice. A)** Schematic illustration of the generation of airway epithelial specific *Taldo1* knockout mice **B-C)** The *Taldo1^fl/fl^* and *Taldo1^fl/fl^* Cre genotype were confirmed by PCR genotyping (**B**), immunofluorescent staining (**C**). **D)** Representative pictures for coimmunostaining of F4/80 or SPC and Taldo1 in lung tissues of mice. N=6 per group. Data are presented as means ± SEMs. **p* < 0.05; ***p* < 0.01; ****p* < 0.001.

**Fig.S3. Airway epithelial-specific knockout of *Taldo1* improves CS-induced airway inflammation and pulmonary remodeling in mouse model. A)** BAL fluid analysis of macrophage counts by flow cytometry. **B)** Expressions of *Cxcl1*, *Cxcl2,* *Ccl2*, *Tnf-α, Mmp12* and *Mmp13* in mouse lung tissues were measured by RT-PCR.**C)** Airway wall thickness was assessed by H&E staining. N=6-8 per group. Data are presented as means ± SEMs. **p* < 0.05; ***p* < 0.01; ****p* < 0.001.

**Fig.S4. *Taldo1* knockout improves CS-induced mitochondrial dysfunction, lung function decline and airway inflammation in mouse model. A-C)** The *Taldo1*^-/-^ genotype was confirmed by PCR genotyping (**A**), western blot (**B**) and RT-PCR (**C**). **D)** Representative images of mitochondrial ultrastructure in mouse lung tissues. **E)** Body weight measurement after CS exposure. **F)** Pulmonary function analysis in the mouse model. **G)** BAL fluid analysis of total cell counts and differential cells by flow cytometry. **H)** ELISA measurement of cytokines in BAL fluid. **I)** H&E staining for mouse lung tissues. **J)** Quantitative analysis of MLI. N=5-8 per group. Data are presented as means ± SEMs. **p* < 0.05; ***p* < 0.01; ****p* < 0.001.

**Fig.S5.** **CS exposure induces mitochondrial dysfunction and inflammation in airway epithelial cells.** A-C) Mitochondrial function was assessed by measuring ATP content (A), mitochondrial membrane potential (B), and mtROS (C) in primary human airway epithelial cells. D-E) mtROS production (D) and mitochondrial membrane potential (E) were evaluated in HBE cells exposed to CSE for 6, 12 and 24 hours using flow cytometry. F-G) OCR (F) and PER (G) were measured in HBE cells treated with CSE for 6, 12 and 24 hours using a Seahorse analyzer. H) Expression of IL-6, IL-8, TGF-β1, CCL2, and MUC5AC in HBE cells exposed to CSE for 6, 12 and 24 hours was measured by RT‐PCR. N=3-5 per group. Data are presented as means ± SEMs. *p < 0.05; **p < 0.01; ***p < 0.001.

**Fig.S6. *TALDO1* knockdown attenuates CS-induced reduction in mitochondrial membrane potential in airway epithelial cells.** *TALDO1* knockdown efficiency was confirmed by western blot (**A**) and RT-PCR (**B**) after siRNA transfection. **C**) Immunofluorescence analysis of mitochondrial membrane potential in HBE cells. N=3 per group. Data are presented as means ± SEMs. *p < 0.05; **p < 0.01; ***p < 0.001.

**Fig.S7. *TALDO1* knockdown attenuates CSE-induced mitochondrial dysfunction in primary human airway epithelial cells. A-B)** mtROS levels in human primary epithelial cells transfected with *TALDO1* siRNA and treated with CSE were detected using a confocal microscope (**A**) and flow cytometry, respectively (**B**). N=3 per group. Data are presented as means ± SEMs. **p* < 0.05; ***p* < 0.01; ****p* < 0.001.

**Fig.S8. *Taldo1* knockout significantly reduced LKU accumulation in response to CS exposure.** A-B) LKU levels in lung tissues (A) and serum from mice (B) were measured using LC‐MS/MS. N=5 per group. Data are presented as means ± SEMs. **p* < 0.05; ***p* < 0.01; ****p* < 0.001.

**Fig.S9. Epa treatment alleviated CS-induced airway wall thickening without causing liver injury as evidenced by H&E staining of airway and liver tissues in mouse model.**

**Fig.S10. Original uncropped images of all western blot membranes.** (**A**) *TALDO1* expression in airway epithelial cells from COPD patients and healthy controls was measured by Western blot. (**B**) Western blot analyses of *TALDO1* in HBE cells exposed to different concentrations and durations of CSE. (**C**) Analysis of TOM20 expression in HBE cells transfected with *TALDO1* siRNA for 24 hours and treated with CSE for additional 24 hours. (**D**) *TALDO1* knockdown efficiency was confirmed by western blot after siRNA transfection. (**E**) TALDO1 expression in CSE-induced HBE cells after MT pretreatment was analyzed. (**F**) TALDO1 expression in mouse lung tissues were measured by Western blot. (**G**)The *Taldo1*^-/-^ genotype was confirmed by western blot.

**References**

1.	L. Van Hoecke, E.R. Job, X. Saelens, and K. Roose, "Bronchoalveolar Lavage of Murine Lungs to Analyze Inflammatory Cell Infiltration," *Journal of Visualized Experiments* no.123 (2017):55398, <https://doi.org/10.3791/55398>.
